# Advances in Techniques for the Structure and Functional Optimization of Therapeutic Monoclonal Antibodies

**DOI:** 10.3390/biomedicines13092055

**Published:** 2025-08-23

**Authors:** Chenchen He, Weijin Huang, Xi Wu, Huanzhang Xia

**Affiliations:** 1 School of Life Science and Bio-Pharmaceutics, Shenyang Pharmaceutical University, Shenyang 110016, China; chench07@outlook.com; 2 State Key Laboratory of Drug Regulatory Science, Division of HIV/AIDS and Sex-Transmitted Virus Vaccines, Institute for Biological Product Control, National Institutes for Food and Drug Control (NIFDC), Beijing 102629, China; huangweijin@nifdc.org.cn

**Keywords:** therapeutic monoclonal antibody, antibody optimization, optimization strategies, computer-aided design techniques, artificial intelligence

## Abstract

Monoclonal antibodies (mAbs), as potent therapeutic agents, have been widely applied in the treatment of various major diseases, including infectious diseases, autoimmune disorders, cancers, and neurodegenerative diseases. However, early-generation mAbs were limited by high immunogenicity, short half-life, and insufficient affinity, which compromised their therapeutic efficacy. With technological advancements, novel approaches such as high-throughput screening and glycosyl modification have been introduced to improve the performance of mAbs. Furthermore, computer-aided design techniques—including molecular docking, molecular dynamics simulations, and artificial intelligence -based methods—are increasingly being employed to accelerate the optimization process. This review summarizes recent progress in the optimization of therapeutic mAbs, with a focus on technological breakthroughs and applications in affinity enhancement, development of broad-spectrum mAbs, specificity modulation, immunogenicity reduction, and stability improvement. Additionally, it discusses current challenges and future directions in antibody optimization. This review aims to provide insights and references for the development and optimization of next-generation antibody drugs, ultimately promoting the clinical application of safer and more effective mAb-based therapies.

## 1. Introduction

Monoclonal antibodies (mAbs) have been widely applied in the treatment of various diseases, including infectious diseases, cancer, and autoimmune disorders. Compared with polyclonal antibodies, mAbs exhibit high purity and high specificity, along with excellent experimental and manufacturing reproducibility under standardized process conditions. In addition, their capacity for sustained large-scale production highlights their advantages across multiple dimensions [1]. As of 2024, more than 120 mAbs drugs have been approved globally for therapeutic use. For instance, Lecanemab and Donanemab have emerged as some of the most effective clinical treatments for early symptomatic Alzheimer’s disease [2,3,4,5], generating significant economic and societal benefits.

mAbs can be obtained through approaches such as hybridoma technology, single B-cell sequencing, and recombinant expression. However, mAbs obtained from initial screening processes often exhibit undesirable features—such as high immunogenicity, short half-life, and insufficient affinity—when applied as therapeutics, thereby limiting their efficacy [6]. Therefore, further optimization is essential to enhance the therapeutic performance, reduce side effects, and improve production efficiency.

With the advancement of genetic engineering technologies, significant progress has been made in mAb optimization strategies. For example, Adalimumab, the first fully human anti-TNF-α antibody approved for clinical use, was derived from a phage display library. Its design retained key binding sites while optimizing peripheral residues to balance affinity and specificity [7]. Trastuzumab adopted a humanization strategy early in its development by grafting the complementarity-determining regions (CDRs) of a murine antibody onto a human IgG framework. This approach aimed to reduce immunogenicity while preserving high specificity for the HER2 antigen [8]. Ixekizumab is a fully human monoclonal antibody targeting IL-17A. It specifically neutralizes the pro-inflammatory cytokine IL-17A without interfering with other cytokines, offering effective control of psoriasis and other autoimmune inflammatory diseases [9]. These antibody drugs have all become first-line clinical treatments.

This review summarizes recent advances in optimization strategies for therapeutic mAbs, with a particular focus on breakthroughs and applications related to improving antibody affinity, broad-spectrum optimization, specificity refinement, reducing immunogenicity, and enhancing stability. The goal is to provide insights and references for future antibody drug optimization, thereby promoting the clinical application of safer and more effective antibody therapeutics. Though this review mainly focuses on the optimization strategies of the structure and function of mAbs, it is equally important to take manufacturability into consideration. Miša Mojca Cajnko et al. performed a series of research and reviews focused on productivity and quality of recombinant antibodies [10,11,12,13].

## 2. Fundamental Principles of Monoclonal Antibody Optimization

**Figure 1:**
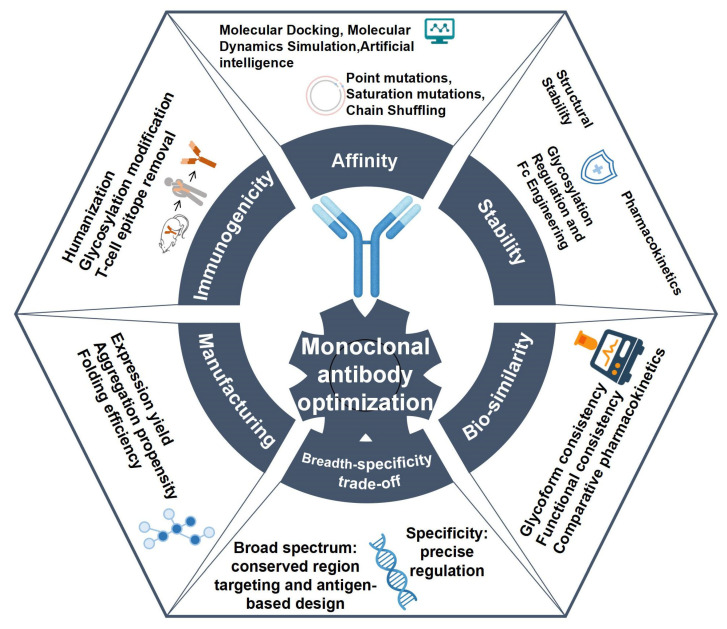
Schematic illustration of monoclonal antibody optimization.

Antibodies screened from natural sources often fall short of meeting clinical demands. To achieve their full therapeutic potential, antibodies must undergo structural and functional optimization across multiple dimensions. Monoclonal antibody optimization is a complex and multidisciplinary process, requiring insights from biology, chemistry, and engineering. Its primary aim is to improve the clinical performance by enhancing antibody affinity, broad-spectrum optimization, specificity refinement, reducing immunogenicity, and improving structural stability [14,15]. An overview of the main aspects of monoclonal antibody optimization is shown in Figure 1.

The main principles of monoclonal antibody optimization include the following key aspects:

Affinity optimization is a critical step in monoclonal antibody development, as it directly affects the antibody’s functional efficiency and therapeutic efficacy in vivo [16]. High affinity ensures effective binding and neutralization of target molecules even at low antibody concentrations, thereby enhancing treatment outcomes and reducing the required dosage. However, antibodies without enough specificity but excessively high affinity can reduce tissue penetration or increase off-target risks, while low affinity may lead to insufficient target engagement [17]. Therefore, precise modulation is essential.

In the development of monoclonal antibody therapeutics, broad-spectrum activity and specificity are competing parameters, and their optimization depends on the disease type, target characteristics, and clinical context. For infectious diseases, rapid viral epitope mutation and immune escape present major challenges, making cross-reactivity against multiple strains or subtypes a top priority [18,19]. By contrast, minimizing the impact on healthy cells is critical for cancer therapy. In these scenarios, antibodies must strictly bind to tumor-specific antigens or overexpressed biomarkers to avoid off-target effects, making specificity the primary concern [20,21].

Immunogenicity optimization aims to minimize the risk of undesired immune responses to mAbs in humans, thereby ensuring safety and clinical applicability. Antibodies derived from non-human species are more likely to be recognized as foreign by the human immune system, potentially inducing antidrug antibodies (ADAs). These can lead to rapid immune clearance, loss of therapeutic efficacy, and even severe adverse reactions [22]. Hence, systematic immunogenicity risk assessment and mitigation at early stages of antibody development is crucial for downstream clinical success.

Stability is a fundamental requirement for the successful translation of mAbs into therapeutic products. Stability refers not only to the antibody’s ability to maintain its structure and function under storage, transportation, and in vitro formulation conditions but also to its resistance to degradation, aggregation, and conformational changes in vivo [23,24,25]. A stable antibody product can achieve a long shelf life, reduced formulation complexity, improved patient compliance, as well as lower immunogenicity and risk of side effects. However, stability is not always synonymous with long-lasting activity, and in certain clinical scenarios, tailored modulation of in vivo persistence may be necessary.

## 3. Strategies for Monoclonal Antibody Optimization

### 3.1. Affinity Optimization

#### 3.1.1. Structure-Based Affinity Optimization Approaches

The introduction of mutations and screening of antibodies through experimental techniques has become a key approach for affinity optimization. A key strategy involves introducing specific mutations to alter the antibody–antigen interaction interface, thereby improving the binding affinity. This strategy encompasses several techniques, including point mutation, saturation mutagenesis, and chain shuffling, which are typically integrated with efficient screening platforms [26]. These methods have been successfully applied to develop potent and safe antibodies for oncology, virology, and the treatment of autoimmune diseases.

Point mutation is one of the most widely adopted engineering techniques for improving monoclonal antibody affinity. This method focuses on the precise substitution of critical amino acids in the antibody’s variable region to enhance binding to the target antigen. When combined with phage display, high-throughput screening enables the selection of antibody variants with superior affinity and specificity. In a study on SARS-CoV-2, site-directed mutations were introduced at key antibody residues to evaluate their effect on affinity. Specific binders against the nucleocapsid protein of SARS-CoV-2 were identified using phage display, and affinity was further enhanced through the combination of multiple beneficial mutations [27]. In another study, Lee et al. [28] screened an anti-ROS1 antibody named 3B20 and performed site-directed mutagenesis on the HCDR3 region. A mutation library was generated by introducing NNK codons at each amino acid position via PCR primers, resulting in multiple variants. Among them, a novel variant 3B20-G1K was selected. Subsequent analysis showed that 3B20-G1K retained high affinity while exhibiting improved specificity, demonstrating stable ROS1 binding even at low concentrations, indicating excellent sensitivity and great clinical potential.

Saturation mutagenesis addresses the limitations of traditional site-directed mutagenesis, such as limited mutational coverage and narrow optimization scope. This technique systematically replaces a selected codon with all possible amino acids, generating a comprehensive antibody library [29]. It significantly increases the likelihood of discovering high-affinity variants and has become a powerful tool in antibody drug development. Researchers have employed high-throughput saturation mutagenesis using microarray-based primer synthesis to generate a primer library with 4370 mutations, constructing a mutant library exceeding 1 × 10^7^ variants. Targeted replacement of specific residues in the original antibody enabled comprehensive affinity optimization against the SARS-CoV-2 spike protein [30].

Chain shuffling is an engineering approach that creates diverse antibody libraries by recombining variable heavy (VH) and variable light (VL) chains. Commonly coupled with phage display or yeast surface display platforms, it is used to enhance affinity and functional diversity. By shuffling the native VH–VL pairing, this method offers operational simplicity with broad mutational diversity, giving it significant potential for affinity improvement. In a study aiming to develop toxin-neutralizing antibodies, Tulika et al. [31] applied light-chain shuffling to optimize an anti-α-cobratoxin antibody, yielding variants with enhanced binding under acidic conditions and improved affinity. In another example, recombination of the heavy chain from anti-ricin antibody 14F11 with the light chain from 9C3 produced a variant with over 200-fold affinity enhancement, demonstrating the powerful role of chain shuffling in affinity maturation [32].

**Figure 2:**
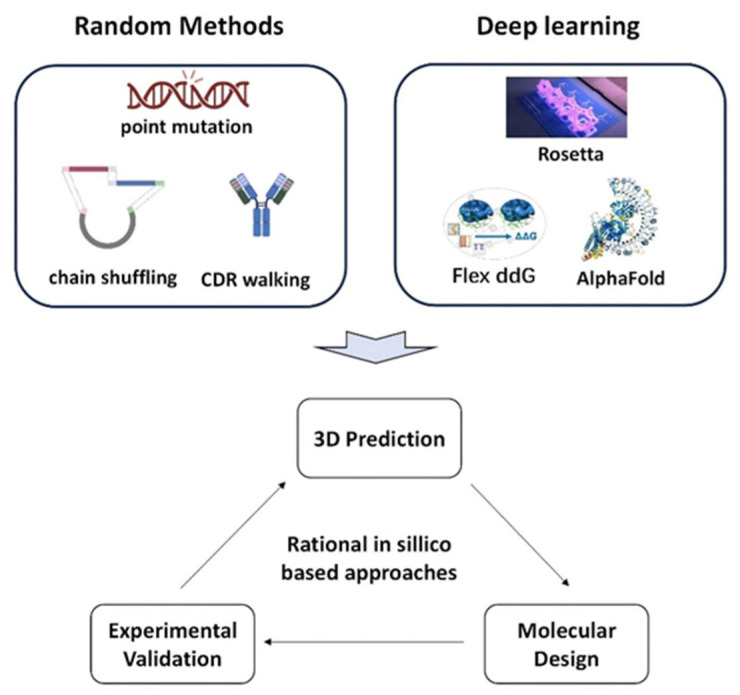
Schematic illustration of experimentally driven and computationally driven pathways for antibody affinity optimization.

In experimentally driven affinity optimization strategies, approaches, such as point mutations at key residues, comprehensive saturation mutagenesis, and chain shuffling, that disrupt natural heavy/light chain pairing have all been successfully employed to construct diverse antibody libraries, achieving significant improvements in affinity across various studies. However, these methods typically rely on high-throughput experimental platforms, involving complex procedures, long screening cycles, and substantial resource consumption. With the advancement of structural biology and computational tools such as artificial intelligence (AI), researchers have increasingly shifted their focus toward structure-informed and algorithm-guided computational design strategies to enhance the efficiency and precision of affinity optimization. Figure 2 illustrates the core workflows and critical components of both experimental and computational strategies in antibody affinity optimization, highlighting a comprehensive path from molecular-level mutations to rational design.

#### 3.1.2. Optimization Approaches Based on Computational Design

With the rapid development of structural biology, AI, and protein engineering, computational design methods have become powerful tools for enhancing the affinity and specificity of mAbs while significantly reducing the workload associated with experimental mutation and screening. These strategies simulate antibody–antigen interactions and predict the impact of mutations on binding interfaces [33], supporting a systematic optimization workflow from structural prediction to rational mutation design.

A prerequisite for effective affinity optimization is a deep understanding of the antibody–antigen interaction interface. Molecular docking serves as a fundamental technique for predicting the three-dimensional conformation of antibody–antigen complexes and identifying key binding residues based on scoring functions that estimate binding energies. In a study of CD133 glycoprotein, researchers employed molecular docking to analyze the scFv (single-chain variable fragment) antibodies against CD133, identifying key residues involved in stable binding and using this information to guide affinity maturation [34]. Similarly, Kunamneni et al. [35] investigated the binding modes of scFv antibodies to the cholecystokinin B receptor, pinpointed critical amino acids, and verified variants with analgesic activity, highlighting the application potential of molecular docking in antibody design and refinement.

Building upon docking algorithms, molecular dynamics (MD) simulations provide insights into the dynamic behavior of antibody–antigen complexes under physiological conditions, revealing structural stability, conformational shifts, and the role of specific residues [36]. These simulations offer critical guidance for predicting binding affinities and designing functional antibodies. For example, in the aforementioned CD133 study, MD simulations were performed alongside docking to assess the antibody-CD133 interactions, revealing a binding interface that remained stable over time, indicative of strong affinity [36]. Similarly, Yang et al. [37] modeled the binding of an antibody to a CFH-derived peptide and observed post-binding structural stability, supporting the conclusion of enhanced affinity.

Further affinity enhancement is often achieved through computational mutagenesis, which involves generating and evaluating multiple mutation combinations to assess their effects on the binding energy to identify optimal variants. In one study combining computational and experimental mutagenesis, Arvind et al. [38] constructed a 3D model of antibody 806 bound to EGFR using docking techniques, evaluated various amino acid substitutions, and experimentally confirmed that certain mutations significantly increased binding affinity. Additionally, high-affinity mutants designed using similar approaches by Desautels et al. as well as Jonniya et al. exhibited improved binding to the SARS-CoV-2 Omicron variant and various EDIII serotypes, respectively [39,40].

In recent years, AI and machine learning techniques have emerged as cutting-edge tools for antibody affinity optimization. AI models can learn from large datasets of antibody–antigen interactions to predict affinity changes, identify beneficial mutations, and even directly generate high-affinity antibody sequences [41,42]. For instance, the AlphaProteo framework of Zambaldi et al. [43] integrates deep learning and molecular modeling, which achieved 3- to 300-fold affinity enhancements across seven protein targets. Prihoda et al. [44] developed the BioPhi platform, which constructs predictive models using data from natural sequence databases and automatically generates high-affinity antibodies. He et al. [45] introduced the PALM-H3 model, capable of designing CDRH3 regions with strong binding to diverse SARS-CoV-2 variants. In addition, Liu et al. [46] created the Ens-Grad method, which considers not only affinity but also dissociation rates and conformational stability, offering a multidimensional approach to antibody optimization.

With the deep integration of structural biology and AI, computer-based antibody optimization has gradually evolved into an integrated platform encompassing a wide range of strategies, including molecular docking, MD simulations, structure prediction, and machine learning. These technologies enable systematic and automated evaluation of mutation impacts on antibody function, allowing for rapid identification of variants with high affinity, low immunogenicity, and excellent stability at the early design stage. With the continued advancement of tools such as AlphaFold and BioPhi, antibody optimization is undergoing a shift from experience-driven to model-driven paradigms, laying a solid foundation for high-throughput, high-precision, and highly efficient development of next-generation antibody therapeutics.

### 3.2. Breadth-Specificity Trade-Off

In antibody design, both breadth and specificity are critical performance metrics, yet they often exhibit a functional trade-off. Breadth requires antibodies to achieve cross-reactivity across antigenic variants, while specificity emphasizes precise and exclusive recognition of the target antigen. Enhancing breadth may reduce the affinity for a single antigenic conformation, whereas improving specificity may compromise recognition of mutated variants. Therefore, when targeting highly variable viruses or tumor antigens, antibody optimization strategies must carefully balance recognition breadth and precision.

#### 3.2.1. Broad-Spectrum Optimization Strategies

When confronting highly variable viruses or heterogeneous tumor-associated targets, traditional mAbs that recognize a single epitope often suffer reduced binding capacity—or even complete loss of function—due to structural changes in the antigen. In such contexts, enhancing the cross-reactivity against multiple antigen variants becomes a central challenge in antibody engineering.

The key to optimizing antibody broad-spectrum activity lies in enhancing structural adaptability and tolerance of antigenic diversity. A commonly used approach is to identify and target conserved structural domains of the antigen to maintain recognition across multiple variants. For instance, Dacon et al. [47] developed a class of mAbs targeting a conserved helical region near the S2’ cleavage site of the coronavirus spike protein, demonstrating broad neutralization against both alpha and beta coronaviruses. Structural analysis revealed that this antibody exhibits significant binding activity across subtypes. This study provided a structural and functional basis for designing fusion peptide-targeting broad-spectrum antibodies, thereby expanding the neutralizing antibody repertoire beyond the receptor-binding domain (RBD)-focused lineages.

Immunogen design is also emerging as a key strategy in broad-spectrum antibody optimization. In an HIV-related study, researchers proposed a structure-guided immunogen design approach. They used MD simulations to analyze antibody-envelope interactions and identified mutations that promote desired antibody mutations crucial for neutralization. By precisely engineering epitope conformations, they selectively activated and drove antibody lineages toward specific mutations that favor the development of broadly neutralizing antibodies (bNAbs) [48]. This approach reshapes antigen presentation at the molecular level and defines antibody evolution trajectories at the systems level, representing a strategic shift from structural engineering to immune modulation [49,50,51].

Thus, the establishment of engineering strategies and immunological programming is not merely conceptual—it encompasses defined molecular targets, structural frameworks, and translational mechanisms that bridge antigen design with lineage-directed immune activation. While broad-spectrum antibodies offer resilience against antigenic diversity, they often face reduced specificity. Balancing breadth and precision under clinically relevant conditions remains a major challenge for next-generation antibody therapeutics.

#### 3.2.2. Specificity Optimization Strategies

Compared with breadth, which emphasizes compatibility with diverse antigenic structures, antibody specificity refers to the exclusive recognition of the target antigen. It serves as a critical safeguard for achieving precision therapy and avoiding off-target toxicity. A natural trade-off often exists between these two properties: enhancing breadth may compromise the resolution of structural differences, while improving specificity can limit the ability to recognize mutated or variant antigens. Therefore, in the treatment of highly variable pathogens or heterogeneous tumors, antibody optimization must dynamically balance structural adaptability and selective recognition.

In addition, specificity is often conflated with affinity, although the two should be clearly distinguished in both biological interpretation and engineering design. Affinity describes the binding strength between an antibody and its antigen, while specificity refers to the selectivity of that interaction. In some cases, antibodies with high affinity may also bind to structurally similar off-target antigens, leading to cross-reactivity or adverse side effects. Thus, focusing solely on affinity enhancement may result in a “high-affinity but low-specificity” pitfall. Achieving truly precise targeting requires fine-tuning of the antigen–antibody interface to reinforce selective binding.

To improve antigen recognition specificity, recent efforts have increasingly shifted from empirical screening to structure-based rational design. Akbar et al. [52] systematically analyzed atomic-level interactions within antibody-antigen complexes and derived a set of local structural motifs predictive of binding patterns. By integrating these motifs with deep learning, they developed a predictive model for antibody specificity. The key finding of this study is identifying a “compact” vocabulary of paratope–epitope interaction motifs. This vocabulary was found to be distinct from motifs involved in general protein-protein interactions, suggesting that the number of antibody-binding motifs is relatively restricted. Monoclonal antibody development can benefit from tuning sequence diversity toward the interaction motifs discovered by the study.

To support these predictive efforts, structural modeling and antigen docking simulations have become essential. Tools such as RosettaAntibody and SnugDock, developed within the Rosetta platform, allow high-precision 3D modeling of antibodies from sequence data and simulate flexible docking with antigens. These tools are particularly effective in modeling conformational adjustments within the CDR-H3 loop and its interaction with epitopes [53]. Krawczyk et al. [54] integrated next-generation sequencing data of immunoglobulin genes (Ig-seq) with the Structural Antibody Database (SAbDab), enabling structural mapping and high-throughput classification of complementarity-determining region (CDR) conformations. This strategy facilitates the rapid identification of potential highly specific binding templates and enhances the translation of sequence data into structural and functional insights. In another study, Xu et al. [55] used the mAb806 antibody as a design template to construct a conformational antigen that mimics an epitope exposed only when EGFR is overexpressed in tumors. This strategy successfully induced the generation of tumor-specific antibodies.

Specificity optimization of mAbs is gradually evolving toward structure-centered rational design and precision recognition paradigms, involving atomic-level contact modeling, flexible docking simulations, structural database annotation, and structure-guided immunogen design. With the continued integration of deep learning and large-scale structural antibody data, specificity engineering strategies are becoming increasingly efficient and predictable, offering a robust molecular foundation for targeted precision therapies.

### 3.3. Reducing Immunogenicity

Early mAbs were primarily derived from murine hybridoma cells. However, the murine sequences of these antibodies are often recognized by the human immune system as foreign, potentially inducing the generation of ADAs. This can lead to rapid loss of drug efficacy and even trigger unintended immune responses. As a result, a variety of strategies have been developed to reduce the immunogenicity of mAbs.

#### 3.3.1. Antibody Humanization

Antibody humanization involves replacing non-human regions with human-derived segments, thereby converting foreign antibodies into forms with reduced immunogenicity that are more suitable for human therapeutic use. Traditional methods include the construction of chimeric antibodies [56] and grafting of the CDR [57].

This strategy has led to the successful development of several marketed monoclonal antibody therapeutics [58,59,60]. For instance, Lecanemab is a humanized IgG1 monoclonal antibody derived from the murine mAb158, designed to target Aβ protofibrils involved in Alzheimer’s disease pathology. Compared to Aducanumab and Gantenerumab, Lecanemab exhibits a 10- to 15-fold higher affinity for Aβ aggregates while maintaining significantly reduced affinity for Aβ monomers. This allows for efficient clearance of toxic aggregates with minimal disruption to normal physiological function [2]. Rozanolixizumab [61,62] is a fully humanized IgG4 monoclonal antibody targeting the neonatal Fc receptor (FcRn). It blocks FcRn–IgG interactions, promoting accelerated IgG degradation and thereby reducing pathogenic IgG levels for autoimmune disease treatment. Its high-affinity design and optimized stability ensure sustained pharmacodynamic activity and safety in vivo.

With the successful clinical application of various humanized antibody therapeutics, the differences among humanization strategies in terms of clinical efficacy, immunogenicity control, and engineering complexity have become increasingly evident. To provide a clearer overview of the core mechanisms and applicable contexts of these strategies, Table 1 summarizes the fundamental principles, advantages, limitations, and representative examples of commonly used humanization techniques.

Although humanization can greatly improve the clinical performance of antibodies, it generally cannot completely eliminate immunogenicity due to residual murine structural elements [65]. To address this challenge, AI and machine learning technologies are being integrated into the humanization pipeline. Deep learning models trained on large human antibody datasets can identify structural and functional patterns in antibody sequences and predict optimal mutagenesis workflows for humanization. A notable example is the YabXnization platform [76], an integrated system combining rational design with AI algorithms, which has demonstrated a high humanization success rate of up to 90%. It is also adaptable to species-specific backgrounds, such as caninization and felinization, making it broadly applicable for multi-species antibody optimization. While enhancing adaptability, new advances have also been made in improving the precision of humanization design. The Hu-mAb tool proposed by the team of Claire Marks further improves the precision of humanization design. Studies have shown high concordance between the model’s mutation suggestions and experimental results—with mutation overlap rates of 68% in the VH chain and 77% in the VL chain. The development of an AI tool significantly reduces the number of required mutations, easing the experimental burden and accelerating the development process [75].

As humanization strategies continue to evolve toward structure-based prediction, algorithm-driven design, and cross-species adaptation, approaches for controlling antibody immunogenicity are also expanding accordingly. In addition to rational replacement of immunogenic residues at the sequence level, glycosylation modifications also play a pivotal role in modulating immune recognition, functional activity, and in vivo behavior, making them an essential and often overlooked approach for reducing immunogenicity.

#### 3.3.2. Glycosyl Modification

Glycosylation is not only a structural modification of antibody molecules but also one of the key regulatory factors influencing their biological activity and pharmacokinetics, particularly within the Fc (constant) region of the antibody. Glycosylation in the Fc region not only affects antibody binding to Fc receptors but also modulates antibody-dependent cellular cytotoxicity (ADCC), complement-dependent cytotoxicity, and overall immunogenicity [77,78]. By optimizing Fc glycosylation patterns, it is possible to finely tune the immune response triggered by antibodies, which holds great promise for the design and production of therapeutic antibodies.

The glycan composition of the Fc region has a significant impact on the in vivo distribution, clearance rate, and half-life of antibodies. For example, a study by Kanda et al. demonstrated that non-fucosylated IgG antibodies with high-mannose-type glycans exhibit enhanced FcγRIIIa binding and ADCC activity. However, these antibodies are rapidly cleared from circulation due to recognition by mannose receptors, resulting in a markedly shortened half-life [79]. In contrast, IgG molecules modified with complex biantennary glycans display improved in vivo stability and extended circulation time. Although their Fc-mediated effector functions may be moderately reduced, they offer superior pharmacokinetic properties overall [80]. This distinction highlights that Fc glycan optimization must carefully balance immune effector functions and in vivo stability and should be guided by clinical objectives through rational design.

Moreover, recent studies have shown that Fc glycosylation can also modulate antibody immunogenicity by altering its spatial conformation and surface charge distribution. Further studies have shown that glycosylation also plays a role in regulating antibody immunogenicity. Research has demonstrated that the addition of small-molecule regulators during mammalian cell culture enables precise control of *N*-glycosylation, thereby avoiding the formation of highly immunogenic glycan structures [81]. This approach helps reduce the risk of immune rejection or adverse reactions in clinical applications. The distribution of glycans on the protein surface can shield key epitopes, thereby minimizing immune responses. Qun Zhou et al. [82] summarized that appropriate *N*-glycan modifications can effectively mask potential T- and B-cell epitopes, reducing the immunogenicity of therapeutic proteins and lowering the incidence of antidrug antibody (ADA) formation.

Glycosylation is not merely a structural modification—it serves as a precise regulatory mechanism for modulating both antibody efficacy and immunogenicity. As such, it holds significant promise in the engineering and industrial-scale production of therapeutic antibodies.

#### 3.3.3. Elimination of T-Cell Epitopes

The elimination of T-cell epitopes relies on the use of bioinformatics tools to predict potential T-cell epitopes within antibody sequences [83], followed by targeted mutations to alter or eliminate these regions. The goal is to reduce interactions between the antibody and the host immune system, i.e., to lower immunogenicity. This approach is particularly important for enhancing the tolerability and long-term efficacy of therapeutic antibodies in vivo, especially for those derived from non-human sources. King et al. [84] proposed a computational epitope deimmunization approach in which amino acid substitutions are introduced at key residues to disrupt the binding between T-cell epitopes and MHC molecules, effectively abolishing epitope recognition. This method involves multiple simultaneous mutations, carefully designed to preserve the functional domains of the antibody—such as the antigen-binding region—without compromising activity or structural integrity. The elimination of T-cell epitopes has become a standard step in the deimmunization design of antibody therapeutics, significantly reducing immunogenicity risk and improving clinical success rates.

However, sequence-level epitope optimization alone does not fully address immunogenicity concerns. In clinical settings—especially under long-term, repeated dosing regimens—the dynamic immune response plays a crucial role in determining the durability and safety of antibody therapeutics. Therefore, assessing the risk of immunogenic responses over extended treatment periods has become an essential step in the development of safer, more effective antibody drugs.

#### 3.3.4. Immune Response Risks in Long-Term Use of Therapeutic Antibodies

In chronic treatment regimens involving repeated administration, therapeutic antibodies as exogenous proteins remain persistently exposed to the host immune system, significantly increasing the risk of ADA induction. This, in turn, can compromise the durability of drug efficacy and clinical safety.

In this context, Vaisman-Mentesh et al. [85] noted that ADA formation is driven by a multifactorial interplay involving antigen presentation efficiency, T and B cell activation, and the breakdown of immune tolerance. Prolonged exposure can enhance T cell-mediated humoral immune responses. Baker et al. further emphasized that ADA generation is also influenced by factors such as antibody structure, aggregation state, and the patient’s immunological background, with antigen exposure duration being a key determinant [86]. Clinical case studies have provided further evidence for the impact of immunogenicity in long-term treatment. For example, Endo et al. reported that TNFSF15 (L1A) genotypes significantly affect long-term responses to anti-TNFα antibodies in patients with Crohn’s disease [87]. Similarly, Lin and Stone described a case in which a patient receiving PCSK9 inhibitors developed ADA-induced acquired resistance after initial therapeutic success, leading to loss of efficacy [88]. These findings underscore the highly individualized nature of immune responses during long-term therapy and their direct impact on treatment outcomes.

To address this risk, Shankar et al. [89] recommended incorporating ADA monitoring and functional assessments throughout the entire treatment cycle in combination with pharmacokinetics (PK)/ pharmacodynamics (PD) analyses to improve proactive intervention strategies. More recently, Sun et al. [90] developed a deimmunization design platform that integrates structural, sequence-based, and machine learning approaches based on T and B cell epitope features, offering a feasible pathway to improve long-term tolerability of therapeutic antibodies.

The immunogenic risks associated with long-term antibody use warrant serious attention. Future efforts should aim to minimize immunogenicity through coordinated strategies involving mechanistic elucidation, individual risk prediction, structural design, and clinical monitoring.

### 3.4. Stability Optimization

#### 3.4.1. Structural Optimization

Structural optimization is one of the foundational approaches for improving the stability of mAbs. Its core lies in modulating conformational properties to enhance protein robustness and folding integrity under unfavorable physicochemical conditions. Among various strategies, disulfide bond engineering has proven to be particularly effective, playing a crucial role in stabilizing the conformations of the variable region, framework region, and antigen-binding loops.

The variable regions, especially the CDRs, are essential for specific antigen recognition, but are also structurally flexible and prone to mutations—making them weak points of the overall molecular architecture. Introducing localized rigidifying mutations or engineering novel disulfide bridges can effectively restrict conformational drift and reduce the aggregation propensity, thus significantly improving thermal and pH stability without compromising binding affinity [91]. Based on MD simulations and experimental verification, Fitriana et al. [92] identified spontaneous structural changes in the antibody light chain as a primary cause of aggregation and deactivation. The introduction of artificial disulfide bonds at key residues successfully locked the optimal light chain conformation, enhancing thermal stability and reducing conformational variability—demonstrating the practical feasibility of structure-guided mutagenesis for stability engineering.

At a more systematic level, Tang et al. [93] proposed a disulfide bond remodeling mechanism based on kinetic modeling, revealing the dynamic reformation of disulfide linkages in antibodies under redox conditions. This study provided theoretical tools for optimizing oxidative environments to enhance folding recovery and stability, offering support for controlled antibody manufacturing. Bozhanova et al. [94] employed structural modeling to discover an endogenous disulfide loop within the HCDR3 region of a neutralizing antibody derived from a hepatitis C virus (HCV)-infected patient. This structural feature not only enhanced the rigidity and thermal stability of the variable region but also maintained—and in some cases improved— the antigen-binding capability, confirming that stability enhancement and functional preservation are not necessarily contradictory at the structural level.

From chain linkage integrity to local conformational rigidity and antigen-binding loop stabilization, disulfide bond engineering demonstrates systemic value in antibody stability optimization. With the integration of computational modeling with high-throughput expression and screening, future structure-based design strategies are expected to advance toward platform-based and predictive stability-driven antibody development approaches.

#### 3.4.2. Modulation of Glycosylation and Fc Engineering

In the stability optimization of therapeutic mAbs, modulation of glycosylation and Fc fragment engineering are regarded as key strategies for extending the in vivo half-life and improving the bioavailability. As a vital functional region of the antibody, the Fc fragment mediates binding to the neonatal FcRn, thereby determining antibody recycling efficiency and clearance rate [95]. Therefore, structural optimization generally focuses on the Fc region, especially through the integration of glycosyl modifications and site-directed mutagenesis, as this significantly prolongs the persistence of antibodies in vivo.

Glycosylation, particularly *N*-linked glycosylation in the Fc region, plays a pivotal role in maintaining the structural stability of antibodies and modulating their interaction with FcRn. Proper glycan composition can reduce nonspecific clearance in vivo while masking potential immunogenic epitopes, thus lowering the risk of ADA formation [96]. Moreover, glycosylation influences antibody-mediated effector functions, such as ADCC and complement-dependent cytotoxicity (CDC). Therefore, fine-tuning glycan patterns is essential for the successful development of therapeutic antibodies.

Targeted mutagenesis in the Fc region provides precise tools for extending the half-life of antibodies. Datta-Mannan et al. [97] introduced mutations such as L234A and L235A to enhance the binding affinity between Fc and FcRn, thereby effectively slowing antibody degradation and significantly increasing in vivo persistence. Similarly, Zhu et al. [98] applied Fc structural modifications in the development of a long-acting antibody against respiratory syncytial virus (RSV), achieving improved in vivo stability and sustained release properties. This antibody demonstrated strong potential as a vaccine alternative.

It is important to emphasize that antibody glycosylation is not solely determined by sequence-level engineering but is also highly dependent on the metabolic environment and process parameters during cell culture. Recent studies have revealed that factors such as glucose concentration, pH, and the accumulation of lactate and ammonia can significantly influence the activity of key glycosyltransferases, thereby modulating the glycan distribution of antibodies [99]. Karst et al. [100] established a semi-mechanistic modeling system within a perfusion bioreactor to quantitatively uncover the intrinsic relationships between glycan profiles and the dynamic changes in nutrients and metabolic byproducts, offering a scientific basis for controlling antibody quality consistency. Furthermore, Okamura et al. [101] introduced the metabolic shift of lactate into cell culture modeling, which markedly enhanced the predictive accuracy for both antibody yield and glycosylation characteristics. These mechanistic models—integrating physiological metabolism with glycosyltransferase activity parameters—present a novel framework for implementing stability-by-design strategies at the upstream process level.

The synergistic application of modulating glycosylation and Fc engineering offers a reliable pathway to develop long-lasting, low-immunogenicity, and highly stable mAbs. Meanwhile, process control strategies based on mechanistic modeling of bioreactors provide a systematic solution for the upstream design of antibody stability and production quality control, serving as an indispensable component in the rational design of antibody durability.

#### 3.4.3. Pharmacokinetic Optimization

As complex macromolecular therapeutics, mAbs rely not only on their high specificity for antigen recognition, but also on favorable PK profiles for clinical efficacy. Optimal PK properties—such as prolonged systemic circulation time, improved bioavailability, and reduced dosing frequency—can enhance therapeutic outcomes while minimizing toxicity and excessive immune activation. This, in turn, can significantly improve patient compliance and quality of life [102]. However, many mAbs still face challenges such as a short half-life, uneven tissue distribution, and rapid clearance, which can compromise both efficacy and safety. Therefore, PK optimization has become an indispensable component of antibody engineering [17].

As an extension of antibody stability, pharmacokinetic behavior reflects the absorption, distribution, metabolism, and excretion (ADME) processes of antibodies in vivo. Optimization strategies are closely linked to structural design and glycosylation patterns, particularly in the Fc region, which strongly influence PK properties. Goetze et al. [103] reported that high-mannose-type glycans in the Fc region accelerate antibody clearance via interaction with mannose receptors. During production, controlling cell culture conditions and glycosylation pathways can effectively reduce the proportion of high-mannose glycans, thereby improving the pharmacokinetics of the resulting antibodies. In related experimental studies, Kanda et al. further validated the dual impact of glycan type on both antibody function and PK properties. While high-mannose, non-fucosylated IgG1 antibodies exhibit stronger FcγRIIIa binding and enhanced ADCC activity, they are rapidly cleared in vivo and have shorter half-lives. In contrast, antibodies modified with complex-type glycans demonstrate slightly reduced effector functions but greater stability and prolonged circulation time [79]. The type of *N*-linked glycan in the Fc region significantly affects antibody stability and clearance rate in vivo. Yu et al. [104] engineered CHO cells to express antibodies bearing Man5-type high-mannose glycans, yielding structurally intact and functionally competent Man5 antibodies. Although these antibodies showed no significant reduction in FcRn binding or antigen affinity, they were rapidly cleared and exhibited shortened half-lives in both mice and rhesus monkeys. This phenomenon was attributed to the Man5 structure being more readily recognized and eliminated via mannose receptor (MR)-mediated pathways.

It is important to note that PK optimization must be precisely tailored to specific clinical requirements. In the treatment of chronic diseases, the primary goal is often to extend antibody half-life, reduce dosing frequency, and enhance sustained therapeutic efficacy. By contrast, for acute infections, toxin exposure, or applications involving immunostimulatory agents, short-acting antibodies or controlled-release strategies may be preferred to prevent immune overactivation or toxin accumulation. Therefore, stability enhancement and PK optimization must be integrated holistically, with antibody engineering strategies customized according to therapeutic context and target characteristics to fully maximize the clinical potential of mAbs.

### 3.5. Computationally Driven Antibody Optimization Technologies

With the widespread clinical adoption of therapeutic antibodies, structural optimization has become a central focus in efforts to improve efficacy, safety, and ultimately, allow successful clinical translation. The preceding sections have systematically reviewed several key dimensions of antibody optimization, including affinity enhancement, breadth tuning, specificity refinement, immunogenicity reduction, and stability improvement—together forming the foundational framework of modern antibody engineering.

Building on this foundation, it is worth emphasizing that the rapid advances in structural biology, molecular modeling, and AI are driving a profound shift from experience-based to model-driven antibody optimization. Computer-aided design has gradually become a critical technological pillar across the entire antibody development pipeline: from structure modeling, binding energy prediction, and immunogenicity scoring to mutation pathway screening and stability assessment, computational tools have significantly improved both the efficiency and precision of antibody design. Table 2 summarizes the current mainstream computational tools and their application cases, providing strategic references for antibody engineering.

## 4. Challenges and Future Directions in Monoclonal Antibody Optimization

Despite the significant progress in monoclonal antibody (mAb) optimization—particularly in enhancing affinity, reducing immunogenicity, improving stability, and refining the pharmacodynamic performance—several critical challenges still limit their practical applications. While high affinity enhances targeting efficiency, it is often accompanied by the risk of non-specific binding, potentially leading to off-target toxicity and adverse immune responses. Therefore, optimization strategies must carefully balance binding strength with specificity modulation. Additionally, although tools for structural prediction and stability assessment have advanced, the accuracy and efficiency of simulating highly flexible regions, such as antibody variable domains, are still limited, constraining the applicability of rational design in complex biological systems.

In addition to structural and functional challenges, the manufacturability of antibody molecules has increasingly attracted attention. Factors such as expression yield, foldability, aggregation tendency, and compatibility with upstream processes often become critical bottlenecks in translating candidate molecules from the laboratory to industrial-scale production. Some antibodies with extremely high affinity or complex structures exhibit poor expression efficiency in CHO cell lines or encounter aggregation and denaturation issues during purification, severely affecting yield and product consistency [10,11,109,110,111]. Therefore, optimization strategies should not only focus on enhancing functional performance but also incorporate manufacturability screening criteria at early development stages to improve downstream efficiency and economic viability.

Building on this foundation, antibody therapeutics must also meet stringent regulatory requirements during clinical translation. Key challenges in large-scale commercial development include ensuring product quality consistency, fulfilling chemistry–manufacturing–control standards, verifying immunogenicity, and demonstrating clinical feasibility [78,112]. These challenges are further intensified in the case of novel antibody formats, such as bispecific antibodies [113] and antibody–drug conjugates [12,114,115], which impose even higher demands on clinical evaluation frameworks and urgently call for systematic regulatory and standardization guidelines.

Despite these challenges, emerging technologies are offering new opportunities for antibody optimization. For example, AI and machine learning models have been widely adopted in key tasks such as antibody structure prediction, affinity modeling, immunogenicity assessment, and mutation design. In particular, deep learning-based protein sequence models have greatly improved the accuracy of 3D structural predictions and post-mutation conformational assessments, offering strong support for multi-objective antibody design [44,46,52,75,106,116,117,118].

On the experimental side, advances in microfluidics, single-cell screening, and automated high-throughput technologies have significantly increased the efficiency and precision of mutant library construction and phenotypic screening, laying a robust technological foundation for identifying optimal antibodies among tens of thousands of candidates [30,46,119,120,121].

Moreover, the landscape of antibody therapeutics is rapidly evolving—from conventional mAbs to more complex formats, such as bispecific antibodies, multi-target fusion antibodies, antibody-drug conjugates (ADCs), and antibody fusion proteins [122,123]. These emerging modalities demand higher standards for affinity, stability, manufacturability and immune regulation, further driving optimization technologies toward greater intelligence, automation, and systematization.

Looking forward, the optimization of mAbs will continue to advance toward smarter, more automated, and more predictive paradigms. AI-driven platforms, combined with systems biology and big data approaches, are expected to replace traditional trial-and-error workflows with closed-loop development pipelines—integrating target discovery, antibody generation, screening, and optimization. Meanwhile, the convergence of immunology and materials science will shift optimization priorities to include antibody delivery systems [124,125], biological stability [126,127], and tissue penetration [128], laying the foundation for broader therapeutic applications. The future of antibody optimization is one of intelligence, biosystem integration, and interdisciplinary innovation—a future where the concept of “designable, predictable, and manufacturable” becomes central to precision medicine.

## Figures and Tables

**Table 1:** The fundamental principles, advantages, limitations, and representative examples of commonly used antibody humanization techniques.

Humanization Strategy	Fundamental Principle	Advantages	Limitations	Representative Example(s)
Chimeric antibody [53]	Fusion of murine variable regions (VH/VL) with human constant regions (CH/CL)	Simple to construct; high expression efficiency	Retains a large portion of murine sequence; high immunogenicity; potential to induce antidrug antibodies (ADAs)	Rituximab (anti-CD20) [63,64]
CDR grafting [57]	Transplantation of murine CDRs onto a human antibody framework	Significantly reduces immunogenicity [65]	Poor framework–CDR compatibility may cause affinity loss and functional impairment	Trastuzumab (anti-HER2) [66]
Structural Modification	Identification of key Vernier residues through structural modeling [67]	Restores/enhances binding affinity	Technically complex and highly structure-dependent	Engineered version of Trastuzumab [68]
Resurfacing [69]	Replacement of surface-exposed non-human residues to reduce immune recognition	Reduces immunogenicity by masking foreign epitopes	Functional regions may be affected, risking loss of activity	Zenapax [70,71]
Specificity-determining residue grafting [72]	Transplantation of only key residues directly involved in antigen binding, rather than full CDRs	Maximally preserves human sequence; further reduces immunogenicity	Unstable binding affinity; requires high-throughput screening; limited current application [73]	HzKR127 [74]
AI-assisted humanization	Deep learning algorithms predict optimal humanization mutations and immunogenic hotspots	High-throughput, automated, and design-accurate	Requires experimental validation to confirm functionality of predicted sites	Hu-mAb [75], YabXnization platform [76]

**Table 2:** Computer-aided methods for antibody optimization.

Category		Tool	Mechanism of Action	Application Example
Affinity	Molecular docking	ClusPro 2.0	Performed molecular docking between the antibody and antigen, analyzed key binding residues, automatically masked non-CDR regions under default parameters, and selected the lowest-energy conformation	CD133 scFv2 successfully bound to D-EC3, and the docked complex reached a stable state after 25 ns [34]
		Rosetta [46]	Modeled the monoclonal antibody–antigen complex and performed structural docking	Obtained the 4E11–DENV1–4 EDIII complex model and validated that single and double mutants exhibited enhanced binding affinity [36]
		Ens-Grad	Identifies key complementarity-determining regions (CDRs) and predicts antibody affinity, optimizes amino acid combinations to enhance the binding ability	Synthesized and tested 12 seed sequences along with 7 Ens-Grad-designed sequences, identifying key Sufficient Input Subset (SIS) residues. The top-performing antibody achieved an EC_50_ of 0.29 nM, showing significantly enhanced affinity compared to ranibizumab [46]
	Molecular dynamics simulations	Rosetta	Performed in silico point mutation simulations on docking models and filtered out conformations inconsistent with experimental results based on predicted changes in binding affinity	Residues E293 and D297 were predicted as key binding sites for mAb806. Simulations indicated that mutations at these positions would abolish binding, and the docking model was subsequently optimized based on this insight [38]
		PALM-H3	De novo generation of artificial antibody heavy-chain CDRs with desired antigen-binding specificity	The generated candidate antibodies exhibited superior binding energies at the interface compared to natural antibodies and demonstrated outstanding neutralization potency against the SARS-CoV-2 Omicron variant XBB, with an IC_50_ of 3.01 μg/L [45]
		AlphaSeq	Constructed an scFv library containing random mutations and measured their binding affinities to the target antigen for model training	The top-performing computationally generated scFv exhibited a 28.7-fold improvement of binding compared to the best scFv obtained through directed evolution [42]
		YabXnization	Traditional CDR grafting and backmutation-driven rational design approaches, alongside AI-assisted integrative computational design strategies	SPR-based binding affinity measurement of the humanized 4-1BB agonist antibody revealed a binding affinity of 4.46 × 10^−9^ M [76]
		AlphaFold 3	Incorporates a substantially updated diffusion-based architecture capable of accurately predicting the joint structure of antibody–antigen complexes	A high-performance structure prediction tool that significantly improves the accuracy of antibody–antigen complex modeling [105]
		AbLIFT	Allows online input of any VH/VL sequence and automatically generates multipoint interface mutation suggestions to enhance affinity	The affinity of D44.1 was enhanced 10-fold, while the dissociation constant of G6des13 was improved approximately 4.5-fold [106]
		RosettaAntibody and RosettaLigand	Generates a large number of docking models to efficiently screen for high-quality binding sites	Determined that the KD value of the mAb for AFB_1_ is in the nanomolar range [107]
Stability	Computational design	Rosetta	Automated design of the heavy–light chain interface to optimize stability	The melting temperature (Tm) of the G6 antibody was increased from 72 to 76 °C [106]
		Flex ddG	Evaluates the impact of mutations on the binding free energy of the protein complex	Binding stability prediction error reduced by 30% [108]
	Structure prediction	Rosetta	Predicts the impact of mutations on the RBD binding free energy to screen variants that balance binding performance and structural stability	The 2130 1 0114 112 antibody showed no significant difference of thermal stability compared to the original clinical antibody COV2 2130, indicating that the optimization process did not compromise thermal stability [39]
Broad-spectrum activity	Structure-based screening	Rosetta	Guided screening strategy to identify broadly neutralizing antibodies with diverse sequences but conserved structural conformations	Broad-spectrum functional immunological assays confirmed that the selected antibodies possess neutralizing activity against multiple HCV variants [94]
Immunogenicity	Humanization	Hu mAb	A random forest classifier built on billions of antibody sequences	The humanness score output by the Hu mAb model is significantly negatively correlated with the experimentally observed immunogenicity of clinical antibodies—i.e., a higher score indicates a lower likelihood of an immune response [75]
		YabXnization	AI-based antibody variable region identification and humanization recommendation platform, integrating structure–sequence databases and immunogenicity assessment tools for multi-parameter optimization	Successfully humanized a murine-derived antibody while preserving its affinity and function and significantly reduced the potential T-cell epitope score [76]
	Structure prediction	BioPhi	Provides a visualized humanization interface with dynamic display of residue substitution suggestions and corresponding changes in humanness similarity scores	Optimization recommendations show high consistency with experimental structures, with 97% of suggested mutations matching actual mutation sites in published humanized antibodies within a benchmark dataset [44]
	T-cell epitope prediction	Rosetta	Designing minimal mutations using a greedy algorithm to disrupt T-cell epitopes while preserving structural stability and functionality	Proposed multiple mutation combinations for sfGFP and exotoxin PE38, effectively eliminating major epitopes while maintaining controllable Rosetta energy changes [84]

## Data Availability

Not applicable.
